# Chiral light intrinsically couples to extrinsic/pseudo-chiral metasurfaces made of tilted gold nanowires

**DOI:** 10.1038/srep31796

**Published:** 2016-08-24

**Authors:** Alessandro Belardini, Marco Centini, Grigore Leahu, David C. Hooper, Roberto Li Voti, Eugenio Fazio, Joseph W. Haus, Andrew Sarangan, Ventsislav K. Valev, Concita Sibilia

**Affiliations:** 1 Dipartimento di Scienze di Base ed Applicate per l’Ingegneria, Sapienza Università di Roma, Via A. Scarpa 16, 00161 Roma, Italy; 2 Centre for Photonics and Photonic Materials, Department of Physics, University of Bath, Bath, BA2 7AY, United Kingdom; 3 Electro-Optics Program, University of Dayton, Dayton, Ohio 45469, USA

## Abstract

Extrinsic or pseudo-chiral (meta)surfaces have an achiral structure, yet they can give rise to circular dichroism when the experiment itself becomes chiral. Although these surfaces are known to yield differences in reflected and transmitted circularly polarized light, the exact mechanism of the interaction has never been directly demonstrated. Here we present a comprehensive linear and nonlinear optical investigation of a metasurface composed of tilted gold nanowires. In the linear regime, we directly demonstrate the selective absorption of circularly polarised light depending on the orientation of the metasurface. In the nonlinear regime, we demonstrate for the first time how second harmonic generation circular dichroism in such extrinsic/pseudo-chiral materials can be understood in terms of effective nonlinear susceptibility tensor elements that switch sign depending on the orientation of the metasurface. By providing fundamental understanding of the chiroptical interactions in achiral metasurfaces, our work opens up new perspectives for the optimisation of their properties.

Optical second harmonic generation (SHG) is a very sensitive technique to characterize the symmetry and morphology of nanopatterned surfaces^1^. It is a background free detection technique that detects magnetic dipole or quadrupole contributions and more importantly a large signal can be produced in macroscopic media lacking inversion symmetry. The interface between two different media is a naturally occurring break in the symmetry as are the asymmetric nanopatterned shapes on metasurfaces. Using laser light to measure a metasurface’s SHG response can easily reveal both of these breaks in symmetry. Another form of symmetry breaking is chirality, the lack of mirror symmetry, which can be uncovered by carefully designed SHG experiments^2^,^3^. The characterisation of surface chirality by SHG experiments has had an enormous impact on the study of chiral molecules that are important for progress in the life-sciences and pharmaceutical industries^4^.

In metal nanopatterned materials the electric field intensity can increase by several order of magnitude due to the mutual effects of field localization near sharp edges (lightning rod effect), on nanoparticles due to localized surface plasmon excitations, and between small metal gaps. Enhanced SH conversion efficiency from a large variety of metal nanopatterned surfaces exploits this effect. Possible planar substrates to exploit the local field enhancement effect include: nano-holes^5^, nano-wires^6^,^7^, and nano-rods^8^. A chiral response can be elicited not only by 3D chiral structures^9^, but also by 2D chiral metasurfaces^10^ or, as theoretically predicted, by 1D chiral elements^11^. The possibility to measure an optical chiral response (or optical activity) with non-chiral elements has also been studied in the past. Optical activity from anisotropic achiral surfaces was reported in ref. 12 based on SHG experiments; despite the achiral material investigated by using specific orientations of the surface with respect to the incident light a chiral response was found. This effect, related to the chirality of the total geometry composed by both the sample and the experimental setup, affects both linear, as well as, nonlinear responses of the sample. However, as stated by the authors, experimental observation of linear CD is more difficult because it is not surface specific and possibly masked by substrate interference. Reference 12 was the first report of the phenomenon, later called, “extrinsic chirality”. With the recent development of artificial materials (metamaterials and metasurfaces) with tailored and enhanced linear and nonlinear response there was a renewed interest in the topic and several papers recently reported experimental linear^13^,^14^,^15^ and nonlinear^16^,^17^ nanostructures characterization revealing extrinsic chirality behaviour.

Here we experimentally and theoretically investigate the effective chiral behaviour of a metasurface formed by self-assembled, tilted gold nanowires (NWs), as shown in Fig. 1a–c. Self-assembly techniques are extremely appealing in order to realize large area nano-structured devices operating in the visible range. They allow the development of highly structured, large area samples without the need for slow and expensive serial fabrication processes. In our experiments the extrinsic chirality is measured by using three different measurement schemes: optical reflectance (OR), photoacoustic absorbance (PA) and second harmonic generation (SHG). Our photoacoustic measurements directly detect light absorbance, verifying for the first time that achiral metasurfaces can act as proper **circular dichroic** materials by selective absorption of circularly polarized light. In these experiments circular polarized light was used to determine the optical chiral behaviour for different orientations between the wires and incident laser beam. As expected, circular dichroism is found for all three methods and it is related to the chirality of the sample plus laser incidence geometry. Indeed we note that for certain NW orientations a non-planar triad is formed by the three directions (Fig. 1d) of i) the wires’ orientation with respect to the surface **f**, ii) the impinging light wave vector **k,** and iii) the normal to the metasurface **n**^13^,^18^. Indeed a non-planar triad of vectors represents a system that has a different handedness when compared to its mirror image; thus the tilted orientation of nanowires on a flat surface as seen from the normal incidence direction is a chiral system. The experimental results produced by the three experimental techniques are in perfect agreement although the nonlinear SHG measurements exhibit a much larger CD factor due to increased sensitivity of the phenomenon on surface properties. Finally the origin of the nonlinear CD was investigated by describing the optical nonlinear response of the metasurface starting from symmetry considerations. Due to the many unknown parameters related to the response of disordered, self-assembled samples, we focused on the main signatures of extrinsic chirality in the nonlinear SHG signal: a) the sign reversal of the circular-difference response when the handedness of the geometry is reversed; and b) CD vanishes when the setup possesses a mirror plane. We report a perfect agreement between our theoretical/numerical analysis and experimental results.

## Results

**Figure 1:**
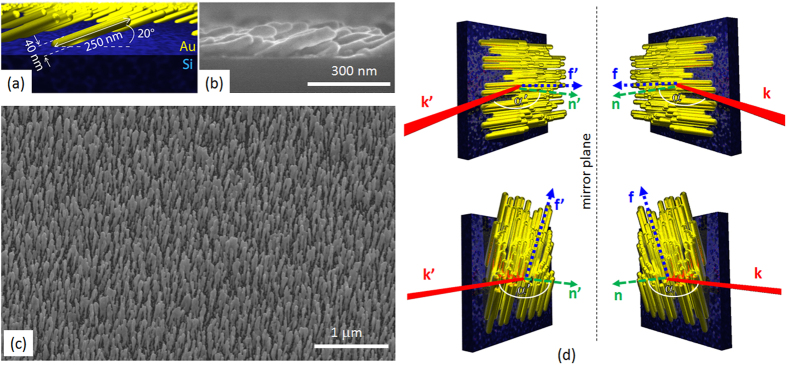
(**a**) Schematic model of the experimental sample with typical geometrical dimensions; (**b**) SEM image of the cross section of the sample, side view; (**c**) SEM image of the sample, top view; (**d**) schematic of the directions involved in the extrinsic chiral behaviour.

The sample of tilted NWs was produced by depositing gold at grazing incidence on a silicon substrate maintained at a temperature of ~300 K. Using this method, also known as the Glancing Angle Deposition (GLAD), a self-ordered forest of Au NWs can be realized as shown in the scanning electron microscope (SEM) images in Fig. 1b,c. The NWs distribution is homogeneous on a 2 cm × 2 cm substrate, the average diameter of the NWs is 40 nm and the average length of the wires is about 250 nm. The gap between wires is approximately 40 nm and they are tilted with respect to the substrate normal by 70°, corresponding to 20° with respect to the substrate plane (see the scheme in Fig. 1a).

**Figure 2:**
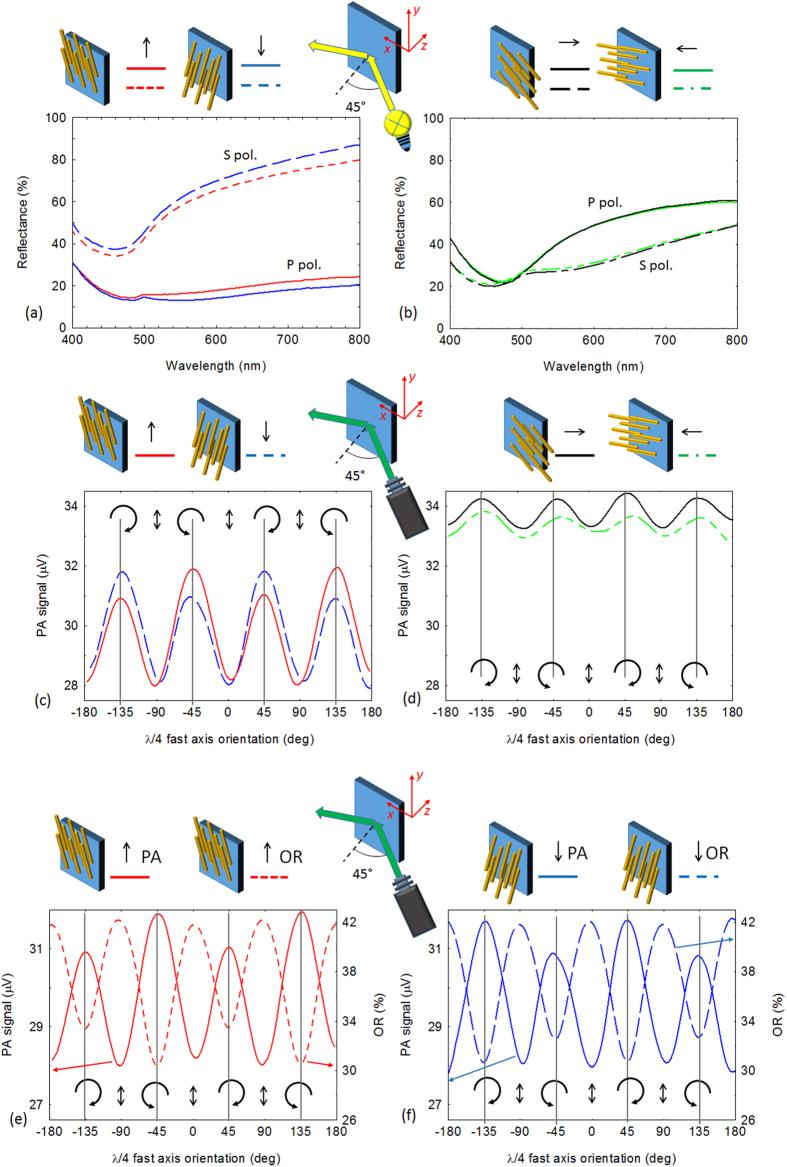
Linear optical measurements. (**a**) Reflectance spectra at a 45° angle of incidence with the wires mainly oriented perpendicular to the plane of incidence, red curve with tip of the wires oriented upward, blue curve downward (solid lines P pol., dashed lines S pol.); (**b**) Reflectance spectra at a 45° angle of incidence with the wires mainly oriented in the plane of incidence, green curve with tip of the wires oriented away from the incident light, black curve with tip of the wires oriented in the opposite direction (solid lines P pol., dashed lines S pol.); (**c**) Photoacoustic signal (PA) at 45° angle of incidence on sample with vertically oriented nanowires, red solid line with wire ends pointing up, blue dashed line for wires oppositely pointed; (**d**) Photoacoustic signal (PA) at 45° angle of incidence on sample with horizontal oriented nanowires, green dashed line with wire ends pointing away the incidence light, black solid line with wires in the opposite direction; (**e**) A comparison of photoacoustic absorbance (PA) signal and optical reflectance (OR) at 45° angle of incidence with nanowires pointing up (PA solid red line, OR dashed red line); (**f**) PA and OR signals with downward wires pointing down (PA solid blue line, OR dashed blue line). On the top of each panel a schematic illustration of experimental sample and laser beam orientation with respect to the nanowire orientations.

The linear reflectance spectra data at an angle of incidence of 45° are plotted in Fig. 2a,b for different spatial orientations of the nanowires and polarization states of the incidence light. It is interesting to observe the linear dichroism caused by the geometric distribution of the wires. With the wires oriented in the vertical direction, we notice high reflectivity for S polarized light and low reflectivity for P polarized light, due to the absorption induced by localized plasmon excitations. As expected, with the nanowires mainly oriented along the horizontal direction the phenomenon is reversed (high reflection with P pol. light and low reflection with S pol. light).

The photoacoustic technique (PA) enables a measurement of the net absorbance of the incident light on the sample by monitoring the heat produced and transferred to the surrounding air. This technique is very useful in all cases in which scattering could affect the signal; even though our sample surfaces are rough the PA technique still captures the absorbed energy. In Fig. 2c,d we report the PA signal measured using a 532 nm laser. With the nanowires oriented in the vertical direction the circular dichroism is evident in the difference of the signal between the two orientations of the quarter-wave plate at −45° and +45°, corresponding respectively to left-handed circularly polarized light (LCP) and right-handed circularly polarized light (RCP). The sign of the chirality is reversed by changing the orientation of the nanowires by 180°, thus indicating the extrinsic nature of the chirality. As expected circular dichroism vanishes when the wires are oriented in the horizontal direction because a non-planar triad is no longer formed between **f**, **k**, and **n**. In this experiment an orientation angle of 0° for the quarter wave plate represents linear S polarized light.

The circular dichroism of the sample, and thus its extrinsic chiral behaviour, was analysed in the linear optical regime by OR and PA again using a 45° angle of incidence; the sample was investigated using a cw laser beam at a wavelength of 532 nm. The OR data was compared using different sample orientations as in Fig. 2e,f. The measurements were made with different incident light polarization states by scanning a quarter-wave retarder plate (see the Experimental Methods section for further details).

In Fig. 2e,f PA measurements are compared to OR for the same sample orientation. The two signals are 180° out of phase because of the energy conservation; for the maximum absorption condition the sample shows minimum reflection, since transmittance through the Si substrate is absent in the visible wavelengths. The difference of the reflectance between left and right-handed CPL, is opposite in sign with respect to the difference in absorbance.

In order to properly quantify the extrinsic chirality of different configurations and to compare the results with nonlinear measurements, we define a normalized circular dichroism parameter in absorbance (nA_CD) defined as^2^,^19^:


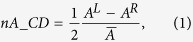


where A^L^ (A^R^) is the absorbance for incoming left-handed (right-handed) circularly polarized light, 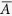 is the average absorbance 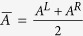 and where we added the factor one-half in Eq. (1) in order to ensure that the nA_CD parameter results are defined in the interval [−1; +1]. The sample oriented with upward wires results in a nA_CD = 0.014.

Reflection and absorption CD behaviours were confirmed by SHG measurements. The experimental apparatus includes a femtosecond-pulse Ti:Sapphire laser source with the central wavelength fixed at 800 nm. SHG was investigated as a function of the input polarization state of the light by scanning a quarter-wave retarder plate (see the Experimental Methods section for details).

**Figure 3:**
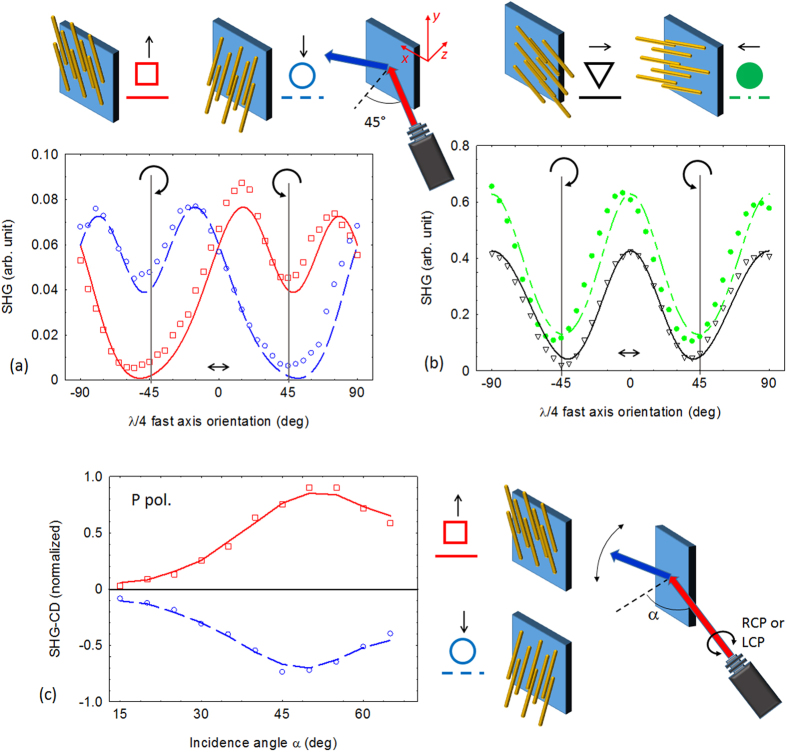
Nonlinear measurements. (**a**) SHG P-pol. signal generated in reflection configuration when the pump field impinges at 45° angle of incidence, the nanowires are oriented as indicated on the top illustrations. The lines are curve fits obtained by using Eq. 9 and the models in the method section; (**b**) SHG signal for sample with nanowire orientations indicated on the top illustrations. The lines are curve fits again obtained from Eq. 9 and the models in the method section. The difference in intensities between the two curves is due to the different Fresnel coefficients (see Eq. 10); (**c**) normalized SHG-CD measurements (see Eq. 2) as a function of the angle of incidence α; (**a**) red squares with nanowire ends pointed up, blue circles with nanowires oppositely oriented. The lines are guides for the eyes.

Figure 3a,b summarizes the results for the SHG signal generated in reflection configuration when the pump field impinges at a 45° angle of incidence, for different sample orientations. In this experiment 0° in the orientation angle of the quarter wave plate represents linear P polarized light. Similarly to PA, the SHG technique reveals that with the wires oriented in the vertical direction the circular dichroism is evident in the difference between the SHG signal for incident RCP and LCP i.e. −45° and +45° orientation of the quarter-wave plate, respectively. The sign of the chirality reverses by switching the direction of the wires from upward to downward, thus indicating the extrinsic nature of the chirality. As expected circular dichroism vanishes when the wires are oriented in the horizontal direction. In order to properly quantify the extrinsic chirality in nonlinear measurements, we define a normalized second harmonic circular dichroism parameter (nSHG_CD) defined as^1^,^2^:


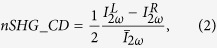


where 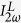 (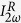) is the second harmonic signal generated by left (right) handed circular polarized pump light, 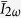 is the average SHG signal 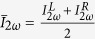 and where, also in this case, we added the one-half factor in Eq. (2) in order to limit the possible values of nSHG_CD to the interval [−1; +1]. The sample oriented with upward wires results in an nSHG_CD = 0.70. The measured nSHG_CD value is 50 times higher than the nA_CD value obtained by PA, validating the improved sensitivity of the second-order optical nonlinear term with respect to the linear characterization^2^,^16^.

We measured the SHG_CD as a function of the incident angle α (Fig. 3c). These results clearly reveal the geometric dependence of the extrinsic chirality, which vanishes when the incidence angle approaches normal incidence as expected by symmetry considerations^16^,^17^,^18^,^20^.

A deeper interpretation of the experimental results can be exposed by analysing the nonlinear optical response of the metasurface through the symmetry properties of its effective second order nonlinear susceptibility tensor. By considering an *x*, *y*, *z* triad oriented as shown in the sample schematics of Fig. 3, the SHG tensor depends on the geometrical orientation of the wires with respect to the incident light:

(i). In the case where the wires are oriented in the vertical direction (*y* direction), the sample has a mirror plane perpendicular to the *x* direction. Therefore, by mirror symmetry in that plane, all the tensor elements with an odd number of *x* components are equal to their own opposite and have to be zero. This yields the following tensor:


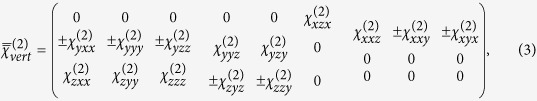


where flipping the sample orientation between upward and downward states, changes the sign of the tensor elements with odd number of *y* components.

(ii). In the case where the wires are oriented in the plane of incidence (*x* direction), the sample has a mirror plane perpendicular to the vertical direction (*y* direction). Therefore, by mirror symmetry in that plane, all the tensor elements with an odd number of *y* components are equal to their own opposite and have to be zero. This yields the following tensor:


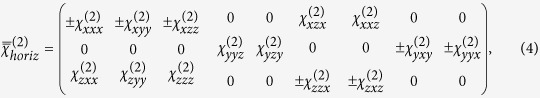


where flipping the sample orientation between leftward and rightward states, changes the sign of the tensor elements with odd number of *x* components.

We note that in both cases the typical chiral elements on the nonlinear susceptibility 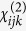 with *i* *≠* *j* *≠* *k* are equal to zero, since the morphology of the sample is not chiral.

The generated P-polarized SH intensity is proportional to the square modulus of the P component of the nonlinear polarization 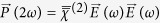 which can be evaluated by matrix algebra. In order to clearly identify the origin of the chiral response, it is suitable to simplify the notation for the tensorial components by adopting a change of the coordinate system, from the *x*, *y*, *z* sample orientations to the coordinate directions defined by the principal light polarizations and the wave vector: *S*, *P*, *k*. The second harmonic intensity can then be written in terms of the *S* and *P* components of the fundamental field as^12^,^21^:


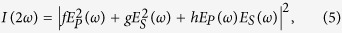


where in general *f*, *g* and *h* are complex numbers, which are linear combinations of the Cartesian susceptibility tensor components 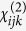. According to ref. 12, for circularly polarized excitation *E*_*P*_(*ω*) = ±*iE*_*S*_(*ω*), thus:


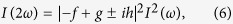


where ± corresponds to right- and left- handed circular polarizations, respectively and *I*(*ω*) is the intensity of the S-polarized component of the pump field. Thus it is clear that CD effects in second harmonic generation occur if (*−f* + *g*) and *h* are simultaneously nonvanishing. We now apply the same argumentations to our experimental conditions and evaluate the effect by considering the nonzero elements of the nonlinear susceptibility corresponding to the two previously described sample orientations. In order to reproduce the SH signal generated in reflection as a function of the rotation angle φ of a quarter wave plate we express the pump field in Cartesian components with respect to the same reference frame used for the tensorial components 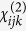. We start with a P-polarized field with an incidence angle α = 45° with respect to the sample normal having components:


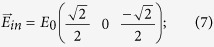


The field passing through a quarter-wave plate with a rotation angle of φ is expressed as:


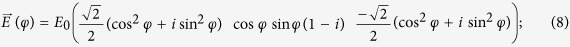


The detected P-polarized SH signal is proportional to the square modulus of the P-polarized nonlinear polarization vector which can be expressed as:


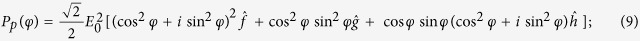


where 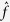, 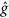 and 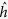 are complex coefficients, analogues to the previously defined ones:


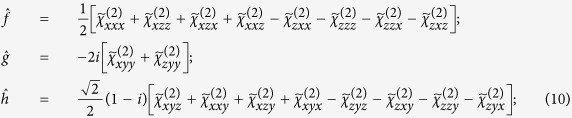


with:


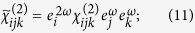


being the Fresnel corrected nonlinear susceptibility tensor components which take into account the pump and second harmonic coupling of free space radiation with the material^12^. Indeed the internal fields 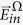, with Ω = ω, 2ω are connected to the free space propagating fields 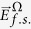
by the expression: 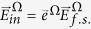. Eq. (10) makes it clear that a unique fit to the tensorial nonlinear susceptibility elements cannot be done for a single set of measurements performed at fixed angle. However the SH signal as a function of incident angles can be retrieved by finding the fitting values for 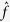, 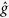 and 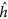 parameters with a least squares method. We also note that for the horizontal orientations of the nanowires (see Equation (4)) all the components of 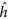 vanish, thus no circular-difference effects are expected and the fit can be performed by using only 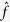, and 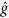 parameters. We verify in Fig. 3a,b that the experimental data are in good agreement with the theoretical calculations obtained by retrieving the 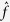, 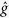 and 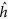 coefficients after applying a least squares parameter fit to the data. Details are discussed in the methods section.

In conclusion we experimentally and theoretically investigate the effective chiral behaviour of a metasurface formed by self-assembled tilted gold nanowires. The effective chirality was confirmed by optical reflectance, photoacoustic absorbance and second harmonic generation measurements. In particular, the photoacoustic technique enabled the direct detection of absorbed light, we verified for the first time that the achiral metasurface acts as an intrinsically chiral material by selective absorption of circular polarised light. Even if the sample presents an achiral morphology, we derived, from symmetry consideration, the effective chiral SHG tensor that was used in order to describe the optical response of the metasurface. The metasurface, due to the high surface to volume ratio and to the artificial chiral response, can be a potential substrate for chiral molecule detection devices. CD measured by SHG shows 50 times higher sensitivity with respect CD measured by linear approaches. Further improvement in the sensitivity can be expected by exploiting the plasmonic resonances present in the proposed structure, by using different laser wavelengths or wires dimensions.

## Methods

### Sample Preparations

The metal deposition for our samples used a generic e-beam evaporation system with a MDC evap-4000 electron gun. The chamber was fitted with a custom-built substrate holder to concurrently support nanowire growth at cryogenic (~100 K) and room (~300 K) temperatures. Liquid nitrogen was allowed to flow through the substrate holder in an open-loop configuration to achieve cryogenic substrate temperatures during the deposition process. Copper and teflon angle blocks with a preset angle of 88° were mounted on the substrate holder. Using mechanical clips, silicon (Si) substrates were attached to the angled surface of the block. Two thermocouples were attached to the angle blocks to measure the substrate temperature during deposition. This experimental setup is discussed in ref. 22. Prime grade double-side-polished (DSP)-type Si <100> wafers were used in this study. Partial wafers were cleaned using acetone, methanol, and isopropyl alcohol followed by nitrogen blow drying. The gold (Au) evaporation pellets were of 99.99% purity from Kurt J. Lesker Company. The chamber base pressure was less than 66 × 10^−6^ Pa prior to the start of the deposition. After the initial preconditioning ramp, the final deposition rate was maintained at 0.3 nm/s. The film thickness was monitored using a SQC-310 deposition controller.

### Linear Reflectance Spectra

The linear reflectance spectra were performed by using a Xe arc lamp in order to generate white light. The light was collected and collimated by achromatic lenses, was polarized in either vertical (*S*) or horizontal (*P*) direction by a film polarizer working in the visible range [400 nm; 800 nm] and then focused on the sample by an achromatic lens with a focal length of 200 mm. The incidence angle of the light was set to 45°. The reflected light was acquired by a large core multimode fiber (500 micron core) and sent to a HAMAMATSU spectral analyzer (working wavelengths [200 nm; 800 nm]).

### Photoacoustic Absorption and Optical Reflectance

The photoacoustic absorption (PA)^23^ and optical reflectance (OR) measurements used a cw, frequency-doubled Nd:YAG green laser emission wavelength at 532 nm and linearly polarized, output beam power of 10 mW with beam spot size diameter of 0.5 mm. The laser light was modulated in time by a chopper at a frequency of 270 Hz. The polarization of the laser light was then changed by rotating a quarter-wave retarder plate in the range [−180°; +180°]. The laser light is incident on the sample at a 45° angle of incidence through a sealed glass window of the photoacoustic cell that encapsulates the sample. In the cell, the sample is surrounded by a 1 mm thick layer of air. When the sample absorbs light, the energy of the light is converted into heat, which is transferred to the small volume of air surrounding the sample increasing the pressure. The modulation of the light intensity, also results in a pressure modulation at the same frequency, thus producing a sound vibration from the cell surface that is collected by a microphone^24^,^25^. At the same time, the reflected light was measured by a standard Si photodiode, enabling the simultaneous retrieval of the reflectance.

### Nonlinear Measurements

The nonlinear measurements were performed by using an amplified femtosecond Ti:Sapphire laser operating at the wavelength of 800 nm. Light pulses are characterized by 150 fs pulse duration, 1 KHz repetition rate and peak power density of about 3 GW/cm^2^. The output light from the laser is *P* polarized, and the measurements were performed as a function of the input polarization state of the light by rotating a quarter-wave retarder plate in the range [−90°; +90°]. The light was incident on the sample at a 45° angle, except for the nSHG_CD measurements, which were performed as a function of angle scanned over the range [15°; 65°]. The SHG signal in reflection was collected by a HAMAMATSU photomultiplier filtered by both an analyzer (that can be set for either P- or S-polarization transmission) and a short-pass filter (<450 nm wavelength) followed by a band-pass filter centered at 400 nm with 40 nm of FWHM. We measured the SHG signal in P pol. state, generated in reflection by a flat gold thick film sample as a reference. The SHG signal generated by the sample is 2.6 times larger than the signal from our reference sample with a flat gold film. The difference is attributed to the nanopatterned morphology of our samples.

### Theoretical Calculations

By considering the non-vanishing elements of the nonlinear susceptibility for a vertically upward orientation of the sample the 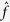, 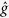 and 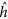 coefficients of Eq. (10) become:


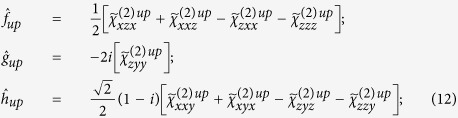


Where, according to Eq. (11), the 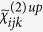 stands for the *ijk*-component of the Fresnel corrected nonlinear susceptibility tensor. Rotating the sample by 180 degrees the orientation of the nanowires becomes downward. In that case some components of the NL susceptibility change sign, according to Eq. (3). We also note that, flipping the orientation, there is no difference between the coupling of light with the sample (as also shown in Fig. 2a,b). It is easy to show that the coefficients 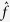, 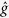 and 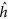 for downward orientation of the nanowires are:


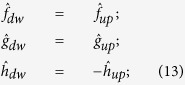


Thus we used the same 3 complex parameters to provide a simultaneous best fit for both SH measurements. For a complete characterization of the nonlinear properties of the metasurface, different sets of measurements at different angles of incidence and under different excitation conditions are required. Indeed there are many parameters contributing to the overall SHG signal and the results emerging from the fitting procedure are not unique. In order to avoid multiple and unphysical solutions form the fit we applied constraints based on the physical response of the system. In principle, the values of the nonlinear susceptibility tensor con be complex quantities. However, being out of the plasmonic resonance, as a first approximation we assume that all the non-vanishing elements of the nonlinear susceptibility are real quantities. With this assumption, according to Eq. (10), fitting parameters are, pure real (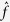), pure imaginary (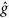) and complex (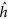) quantities, respectively. With these constraints, a qualitative analysis con be performed by normalizing both experimental and numerical quantities to their average values and by introducing some constraints on the coefficients based on physical approximations. Since the measured SHG signal is proportional to the square modulus of the P-component of the nonlinear polarization defined in Eq. (9), normalization of the coefficients allows a comparison between the behaviours of the two quantities as a function of the quarter wave plate. The least squares routine then retrieves the best normalized parameters that reproduce the behaviour of the SHG signal as a function of the quarter wavelength plate. Graphical results comparing experimental data and theoretical fit obtained under the previous assumptions are shown in Fig. 3a. The relative standard deviations on the normalized fitting parameters are lower than 5%.

Small discrepancies of experimental data with respect to the symmetric theoretical results with respect to φ = 0 are due to possible local misalignment of nanowires with respect to the vertical direction.

A different approach is required for horizontal orientation of the nanowires. Although the components of the NL susceptibility with odd number of *x-*components change sign when rightward or leftward orientation of the wires is considered, according to Eq. (4), the light coupling is different for the two horizontal orientations. For example we have: 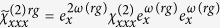, for right orientation and 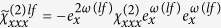 for left orientation.

Thus, being 
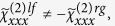
 due to different linear coupling of fields with the sample for opposite orientations of the nanowires, we cannot take full advantage, in this case, of the symmetry properties of the NL susceptibility tensor. However, by substituting the non-zero elements of Eq. (4) into Eq. (10) we obtain, for right oriented nanowires:


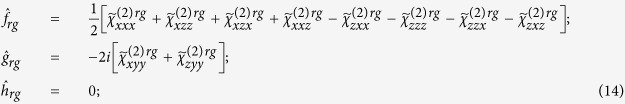


while, for left oriented nanowires the parameters are:


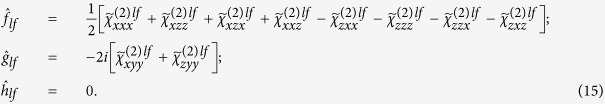


Thus the SH measurements corresponding to horizontal orientation of the nanowires have been fit by two independent parameters for each case. According to the discussion performed for the previous case, in order to limit the possible cases, we assume the fitting parameters to be pure real (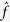) and pure imaginary (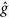). Following the previously described procedure we obtain the theoretical behaviour shown in Fig. 3b with a relative standard deviation for the normalized parameters lower than 2%. As expected there is no extrinsic chiral behaviour (Fig. 3b). Also in this case we notice small discrepancies in the symmetry of the SH signal with respect to the φ = 0, possibly due to small local misalignments of the nanowires.

## Additional Information

**How to cite this article**: Belardini, A. *et al*. Chiral light intrinsically couples to extrinsic/pseudo-chiral metasurfaces made of tilted gold nanowires. *Sci. Rep.* **6**, 31796; doi: 10.1038/srep31796 (2016).
